# Detecting Loci under Recent Positive Selection in Dairy and Beef Cattle by Combining Different Genome-Wide Scan Methods

**DOI:** 10.1371/journal.pone.0064280

**Published:** 2013-05-16

**Authors:** Yuri Tani Utsunomiya, Ana Maria Pérez O’Brien, Tad Stewart Sonstegard, Curtis Paul Van Tassell, Adriana Santana do Carmo, Gábor Mészáros, Johann Sölkner, José Fernando Garcia

**Affiliations:** 1 Departamento de Medicina Veterinária Preventiva e Reprodução Animal, Faculdade de Ciências Agrárias e Veterinárias, UNESP - Univ Estadual Paulista, Jaboticabal, São Paulo, Brazil; 2 Division of Livestock Sciences, Department of Sustainable Agricultural Systems, BOKU - University of Natural Resources and Life Sciences, Vienna, Austria; 3 Departamento de Apoio, Saúde e Produção Animal, Faculdade de Medicina Veterinária de Araçatuba, UNESP - Univ Estadual Paulista, Araçatuba, São Paulo, Brazil; 4 Bovine Functional Genomics Laboratory, ARS-USDA - Agricultural Research Service - United States Department of Agriculture, Beltsville, Maryland, United States of America; University of Queensland, Australia

## Abstract

As the methodologies available for the detection of positive selection from genomic data vary in terms of assumptions and execution, weak correlations are expected among them. However, if there is any given signal that is consistently supported across different methodologies, it is strong evidence that the locus has been under past selection. In this paper, a straightforward frequentist approach based on the Stouffer Method to combine *P*-values across different tests for evidence of recent positive selection in common variations, as well as strategies for extracting biological information from the detected signals, were described and applied to high density single nucleotide polymorphism (SNP) data generated from dairy and beef cattle (taurine and indicine). The ancestral *Bovinae* allele state of over 440,000 SNP is also reported. Using this combination of methods, highly significant (*P*<3.17×10^−7^) population-specific sweeps pointing out to candidate genes and pathways that may be involved in beef and dairy production were identified. The most significant signal was found in the *Cornichon homolog 3* gene (CNIH3) in Brown Swiss (*P* = 3.82×10^−12^), and may be involved in the regulation of pre-ovulatory luteinizing hormone surge. Other putative pathways under selection are the glucolysis/gluconeogenesis, transcription machinery and chemokine/cytokine activity in Angus; calpain-calpastatin system and ribosome biogenesis in Brown Swiss; and gangliosides deposition in milk fat globules in Gyr. The composite method, combined with the strategies applied to retrieve functional information, may be a useful tool for surveying genome-wide selective sweeps and providing insights in to the source of selection.

## Introduction

Selection changes the frequency of advantageous variants and their neighbor polymorphic sites, sweeping the genome and leaving patterns that become prevalent in a population despite chromosome recombination [1]. These patterns are broadly referred as signatures (or footprints) of selection, and many methods have been developed for identifying them from genomic data [2]. The application of such approaches to dairy and beef cattle can help detecting chromosome regions that underwent not only natural but also anthropogenic selection, and that may be associated with traits of economic interest.

The available portfolio of methodologies varies in terms of the underlying selection processes assumed, the age of the sweep, and if the test is performed within-population or depends on population comparisons (**Table 1**). In this scenario, one may expect that correlations among different tests are weak. However, if there is any given signal consistently supported across different methodologies, it may be strong evidence that the locus has been under past selection.

**Table 1:** Types of Signatures of Selection detectable from genomic data. Ages of selection are based on estimations for human data in years, assuming a generation interval of 25 years [2].

Type of signature	Detectable pattern	Methodologies	Underlying selection phenomena	Population level	Age of selection (generations)
Function-altering mutation	Changes in non-synonymousto synonymous variation ratio in the open reading frame of a coding region	*ω = D_n_/D_s_* [3]	Positive and Purifying selection	Within species	>40,000
Local genetic diversity depression	Deficit of local heterozygosity compared to the rest of the genome	*ZHp* [4], SNP heterozygosity [5]	Positive selection	Within populations	<10,000
Change in the allele frequency spectrum	Increase in the frequency of derived alleles	*ΔDAF* [6], *Tajima’s D* [7], *Fu and Li’s* *D-test* [8], *Fay and Wu’s H-test* [9],*CLR* [10]	Positive selection	Within and between populations	<3,200
Populationdifferentiation	Difference in the allele frequencies between populations	*F_ST_* [11]	Positive and Balancing selection	Between populations	<3,000
Extended haplotype homozygosity	LD persistency and unusual long-range haplotypes	*LRH* [1], *iHS* [12], *XP-EHH* [13],*Rsb* [14], *ΔiHH* [6], *varLD* [15]	Positive selection	Within and between populations	<1,200

Recently, Grossman et al. (2010) [6] stated that “*If each signature provides distinct information about selective sweeps, combining the signals should have greater power for localizing the source of selection than any single test*”. Driven by this thought, they developed a Bayesian method for combining *P*-values from different approaches, namely Composite of Multiple Signals (CMS), which was capable to discriminate causal variants from neutral markers in simulated data. Application of CMS to real data led to the discovery of evidence of recent positive selection in LARGE and IL2 in Nigeria human population, genes that were previously incriminated in resistance to Lassa Fever [16].

Although suitable for analysis of human populations, CMS is still challenging to be applied to cattle genomic data, as the computation of likelihood tables requires coalescent simulations using calibrated demographic models in an attempt to mimic the empirical data. Despite availability of good models for cattle history [17], uncertainties around the model and specific recent events that happened during breed formation makes difficult matching the simulations to the real data.

This paper describes and applies to dairy and beef cattle data a straightforward frequentist meta-analysis approach for combining *P*-values across different tests for footprints of recent positive selection in genome-wide single nucleotide polymorphism (SNP) data, targeting common, moderate frequency variants. Two between and two within population tests for selection sweeps are covered, divided into three different categories: extended haplotype homozygosity (*EHH*), change in the allele frequency spectrum and local heterozygosity depression. Strategies for assigning relevant SNP to genes are also described, allowing for exploration of the biological meaning of the findings and facilitating hypothesis generation. Additionally, the ancestral *Bovinae* allele state of over 440,000 SNP is reported.

## Materials and Methods

### Samples and Quality Control

Genotypes for Illumina® BovineHD Genotyping BeadChip assay of Angus (ANG), Brown Swiss (BSW), Gyr (GYR) and Nellore (NEL) individuals were available for prospection of selection sweeps. Details on sample size and data source for each breed can be found in **Table 2**. Only autosome markers (n = 742,910) were included into the analyses. SNP were removed from the dataset if they did not exhibit: 1) minor allele frequency (MAF) greater than or equal to 0.03, 2) *P*-value for Hardy-Weinberg Equilibrium (HWE) greater than or equal to 1×10^−6^ or 3) Call rate (CR_SNP_) greater than or equal to 90%. After the SNP quality control (QC), individuals exhibiting call rate (CR_IND_) below 90% were also removed. This procedure was performed for each breed genotype’s dataset in parallel using *PLINK* [18]. In order to mitigate relatedness in the dataset, individuals were further investigated for the proportion of alleles shared identically by descent using *PLINK*. Potential parent-offspring, half-siblings and duplicate pairs were conservatively removed (see **File S1** for details). SNP commonly passing QC in all four breeds were then overlapped. As the final SNP set consisted of markers passing QC with relatively small amount of missing data, and most of the methods for the detection of selection sweeps do not accommodate missing values, an imputation procedure was adopted to fill the existing missing genotypes. For this purpose, *fastPHASE* software was used [19] with the following arguments: *-H-4 -K10 -T10 -C25*.

**Table 2:** Description of cattle genotypes available for analysis before (BF) and after (AF) filtering for cryptic relatedness and quality control.

Breed	Code	Subspecies	Purpose	HapMap^a^		BOKU^b^		ZGC^c^		Total	
				BF	AF	BF	AF	BF	AF	BF	AF^f^
Angus	ANG	*Bos taurus*	Beef	27	24	0	0	0	0	27	24
Brown Swiss	BSW	*Bos taurus*	Dairy	24	13	48	31	0	0	72	44
Gir	GIR	*Bos indicus*	Dairy	30	23	0	0	0	0	30	23
Nellore	NEL	*Bos indicus*	Beef	35	24	0	0	691	21^d^	726	45

a The Bovine HapMap Consortium [29].

b University of Natural Resources and Life Sciences, Vienna.

c Zebu Genome Consortium.

d The actual number of NEL samples passing control criteria was 581: 557 for ZGC and 24 for HapMap. In order to avoid an unbalanced dataset, we decided to keep a final set of 45 NEL: all 24 HapMap samples plus 21 randomly chosen ZGC samples.

f Final base dataset used for the selective sweep analyses.

### Ancestral Allele Discovery

Since some methodologies for detecting positive selection rely on the comparison of the recombination breakdown between haplotypes carrying the ancestral and the derived allele [1], [12], [13], ancestral allele states were assessed using outgroup species assumed to be derived from a common founder *Bovinae* species that included 2 Gaur (*Bos gaurus*), 6 Water Buffalo (*Bubalus bubalis*) and 2 Yak (*Bos grunniens*) with genotypes derived from the same assay. Genotypes for the three outgroup *Bovinae* species were pooled into a single dataset. Markers with a CR_SNP_ of 100% (i.e. the SNP probe designed to hybridize bovine DNA also recognizes other *Bovinae* species, meaning that the target sequence is within a syntenic block across the outgroups and may have been inherited from a common ancestor) and MAF = 0 (i.e. monomorphic markers, being the one single allele present likely to be the common ancestral variant) were sought. For each case, the major allele (frequency = 100%) was determined as ancestral. The final SNP set was then defined and included markers passing QC with ancestral allele information available.

### Genome-wide Scan Methods for Positive Selection

#### Long-range haplotype based methods

The two methodologies described here are based on the concept of Extended Haplotype Homozygosity (*EHH*) [1], and were applied using the *rehh* package in *R* [20] with minor adaptations to the source code. As the basis for the two tests, the integrated *EHH* for the ancestral allele (*iHH_A_*), derived allele (*iHH_D_*) and SNP site (*iES*) was calculated for each marker. *EHH Method 1*: Voight *et al.* (2006) [12] described a within population score for the ratio between *iHH_A_* and *iHH_D_*, called Integrated Haplotype Score (*iHS*):
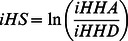



As *iHS* distribution is approximately normal, the scores are divided into 20 equally sized bins according to their derived allele frequencies, and then standardized to have mean 0 and variance 1. The scores reflect how unusual the haplotypes containing the ancestral (positive values) and derived (negative values) allele are, relative to the entire genome. As both tails from the distribution were of interest, two-sided *P*-values were derived as 1–2|φ(*iHS*)−0.5| from the Gaussian cumulative density function. *EHH Method 2*: Tang *et al*. (2007) [14] defined *Rsb*, a between populations test, as:
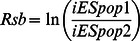



The outcome also resembles a normal distribution. Unlike *iHS*, the standardization procedure recommended by [14] does not divide scores into bins and uses the median instead of the mean. Positive values suggest selection in the population used in the numerator, while negative values indicate signals in the population used as denominator. For each pair of breeds, *Rsb* scores were calculated using the standardization procedure recommended by Tang *et al*. (2007) [14]. As every population was used both as numerator and denominator, one-sided upper tail *P*-values were derived from the normal cumulative density function.

#### Change in the allele frequency spectrum based method

Grossman *et al.* (2010) [6] described a simple method based on the difference in the derived allele frequency between populations (Δ*DAF*). Values range from −1 to 1 and are normally distributed. Δ*DAF* scores were standardized using the distribution’s mean and standard deviation, and one-sided upper tail *P*-values were obtained.

#### Local heterozygosity depression based method

Rubin *et al*. (2010) [4] defined and applied a *Z*-score test for local heterozygosity depression (*ZHp*) on whole genome sequence data of domestic chicken, which basically expresses how much the expected heterozygosity in chromosome windows deviate from the average genome heterozygosity. The approach was adapted to each SNP site and computed using the observed instead of the expected heterozygosity values. The values were standardized to produce mean 0 and variance 1. For this method, negative values were of interest and the resulting site heterozygosity scores were multiplied by −1 in order to switch their direction, yielding a new statistic called *SHp* (i.e. site *ZHp*). One-sided upper tail *P*-values were obtained for each score.

### Meta-analysis of Multiple Tests

As all applied methodologies had *P*-values retrieved from normal distributions with same parameters (mean 0 and variance 1), the weighted version of Stouffer method was adapted for the combination of *Z*-transformed *P*-values, as reviewed by [21]. For each marker and each test *i*, the respective *P*-value was transformed into a *Z*-score by *Z*_i_ = −φ^−1^(1−p_i_). Within population tests were performed only once per breed, hence their respective weight *ω_i_* was set to 1. For each comparison of between population tests, the *Z*-score was weighted to 1/*n*, where *n* is the number of comparisons. Then, the combined statistic of *k* tests, for each SNP in each breed, was defined as:


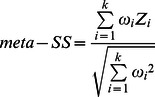


The *meta-SS* (stands for Meta-analysis of Selection Signals) scores were referred back to the standard normal distribution in order to obtain combined significance values, which were intended to address either the combination of information among different, independent tests can reject the shared null hypothesis (neutral marker). Significance level for genome-wide *meta-SS P*-values was based on a Bonferroni threshold (α = 0.05/n_SNP_).

### Functional Annotation

For every peak crossing the significance line, three different strategies for the annotation of functional features were applied, based on the genomic coordinates from the UMD3.1 assembly [22]. *Strategy 1*: Since any given gene harboring signals is a direct candidate, the presence of significant intragenic SNP was checked in the *Ensembl Variation 67* database using the BioMart tool [23]. *Strategy 2*: The closest gene in the vicinity of the most relevant SNP of a given peak could be responsible for the signal. Hence, the most significant SNP from each observed peak was isolated and the closest gene to it was mapped using the *ClosestBed* algorithm from the *BedTools* software [24]. *Strategy 3*: Since there are cases where variants from multiple genes in linkage disequilibrium (LD) with the marker contribute to the signal together, due to the fact that functionally related genes are often spatially close to each other [14], the third approach was based in a window scheme to capture genes that were potentially in LD with the significant SNP. The derived gene lists were processed in *DAVID* [25]–[26] for annotation of functional terms. Although *DAVID* provides means for enrichment analysis, with significance tests for overrepresented terms, the inclusion criteria of functional terms was solely based on existence of information. Finally, the *Enrichment Map Cytoscape plug-in* [27] was used to build networks of inter-related terms based on the number of genes shared between terms, i.e., no hypothesis or significance test was applied, being the networks strictly descriptive. Terms were drawn as nodes (circles). Edges linking nodes represented gene sharing, and their thickness, the degree of gene set overlap (i.e., proportional to the number of genes being shared). An extended description of this section is provided in **File S1**.

## Results

### Ancestral Allele Discovery

By assessing the outgroup species genotypes, an average CR_IND_ of 83.79%, 96.93%, 94.87% and 88.63% for Water Buffalo, Yak, Gaur and pooled data was observed, respectively. From the initial set of 742,910 autosome markers, considering only markers perfectly typed across the pooled outgroup samples (CR_SNP_ = 100%), a total of 559,663 SNP probes were successfully hybridized (71.94%), and 111,376 SNP were polymorphic (MAF>0). Hence, a total of 448,287 SNP (56.75%) had their ancestral allele determined, being provided as a TSV file (**File S2**).

### Quality Control

Number of SNP passing QC was 579,470, 554,826, 485,655 and 461,702 for ANG, BSW, GYR and NEL, respectively. Overlapping of the four SNP lists retrieved a final set of 281,994 markers, from which 157,702 had ancestral allele information available. Even with the drastic drop in the number of SNP, the intermarker distance mean and median were 15.94 kb and 6.43 kb, respectively, superposing the median spacing of 37 kb declared for the BovineSNP50 assay [28]. These findings indicated that the overall marker coverage was satisfactory, although generation of local gaps by QC was observed. No individuals were removed due to QC. The number of remaining samples for each breed, after duplicates and first degree relationship removal, was: 24 for ANG, 44 for BSW, 23 for GYR and 581 for NEL. As NEL exhibited a sample size much larger than the other breeds, 45 individuals were sampled from the total (details in **File S1**). Details on the final base dataset used for all further analyses can be found in **Table 2**.

### Identification of Selection Signals and Functional Annotation

**Figure 1:**
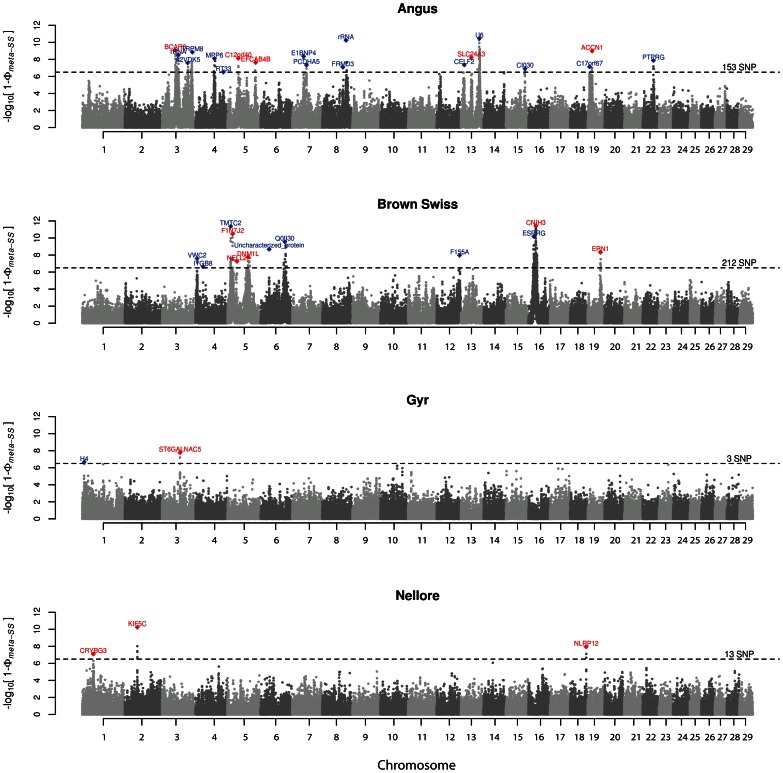
Manhattan plots of genome-wide *meta-SS* –log10(P-values) for Angus, Brown Swiss, Gyr and Nellore breeds. Number of SNP indicated represents count of markers crossing the significance line (*P*<3.17×10^−7^). Red and blue diamonds are intragenic and intergenic top SNP on peaks, respectively.

All performed tests for footprints of selection resembled a normal distribution (**Figure S1**) and genome-wide *Z*-transformed *P*-values were weakly correlated (**Figure S2**), satisfying the independence condition for meta-analysis. Genome-wide distribution of *meta-SS P*-values and the closest genes to the top of the peaks can be found in **Figure 1**. The number of SNP with *P*-value crossing the genome-wide significance (*P*<3.17×10^−7^) was: 153 for ANG, 212 for BSW, 3 for GYR and 13 for NEL. The most significant SNP was found in BSW (P = 3.82×10^−12^), and is an intronic variation in *Cornichon homolog 3* gene (*CNIH3* - ENSBTAG00000044171), located at BTA16:28478192.

**Figure 2:**
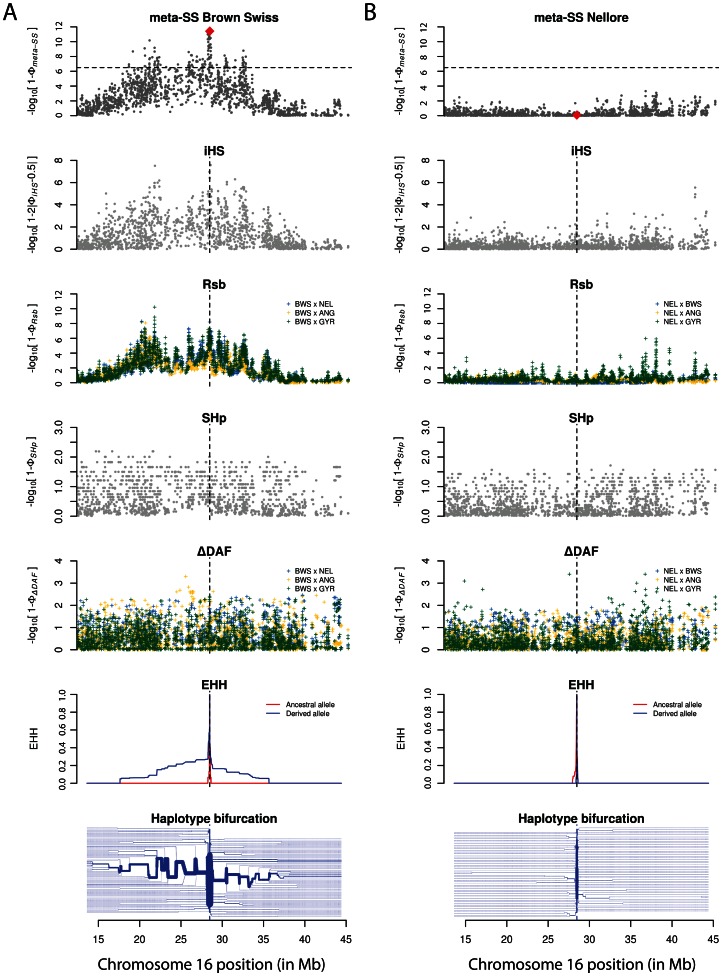
*meta-SS*, component tests, EHH and derived allele bifurcation for CNIH3 in Brown Swiss (A) and Nellore (B). Vertical dashed lines and red diamonds represent the position of the intronic SNP detected as highly significant in Brown Swiss (BTA16:28478192, P = 3.82×10^−12^). Horizontal dashed lines mark the Bonferroni significance threshold (P<3.17×10^−7^).

In order to illustrate the potentiality of combining signals resulting from different methodologies for the detection of positive selection, a regional plot of *P*-values for each of the individual tests for the CNIH3 region in BSW (candidate for being selected) and NEL (candidate for being neutral) was provided in **Figure 2**. For the same genomic region, two extra graphics were provided: 1) a EHH decay plot, showing the decrease of the probability of identity by descent as a function of the distance from the core SNP site (i.e., the *CNIH3* intronic SNP) for both the haplotypes containing the derived and ancestral alleles, and 2) a bifurcation diagram for the haplotypes containing the derived allele, representing the breakdown of LD at increasing distances from the core allele (in this case, the derived allele) at a given core SNP (in this case, the *CNIH3* intronic SNP). It can be seen from the BSW and NEL comparison that the signal of the unusual derived allele long haplotype in BSW, revealed by the *meta-SS* statistics, is not detectable in NEL. It is noticeable, by the shape of the SNP significances distribution in the *meta-SS* scatter plot, that *iHS* and *Rsb* had higher influence in the composite test, and the combination of methods penalized SNP with little statistical support.

The number of genes directly harboring significant SNP was 20 for ANG, 27 for BSW, 1 for GYR and 3 for NEL (the full list can be viewed in **File S3**). Two synonymous exonic SNP for ANG and BSW, one non-synonymous variation (BTA7:42652319, Ala->Thr) for a gene of the olfactory receptor family (*LOC524290*/*OR2W3* - ENSBTAG00000025293) in ANG (*P* = 7.65×10^−9^) and a 3′UTR variation (BTA2:47315215) for the *KIF5C* (*kinesin family member 5C* - ENSBTAG00000018125) gene in NEL (*P* = 2.68×10^−7^) were found. All other variants within genes were located in introns. The application of the LD-window approach (*Strategy 3*) retrieved SNP windows with an average size of 576.8 kb overall breeds, and the largest window spanned 1.83 Mb. Total number of genes within windows included in each breed specific list was: 309 for ANG, 177 for BSW, 4 for GYR and 14 for NEL (full lists can be found in **File S4**).

**Figure 3:**
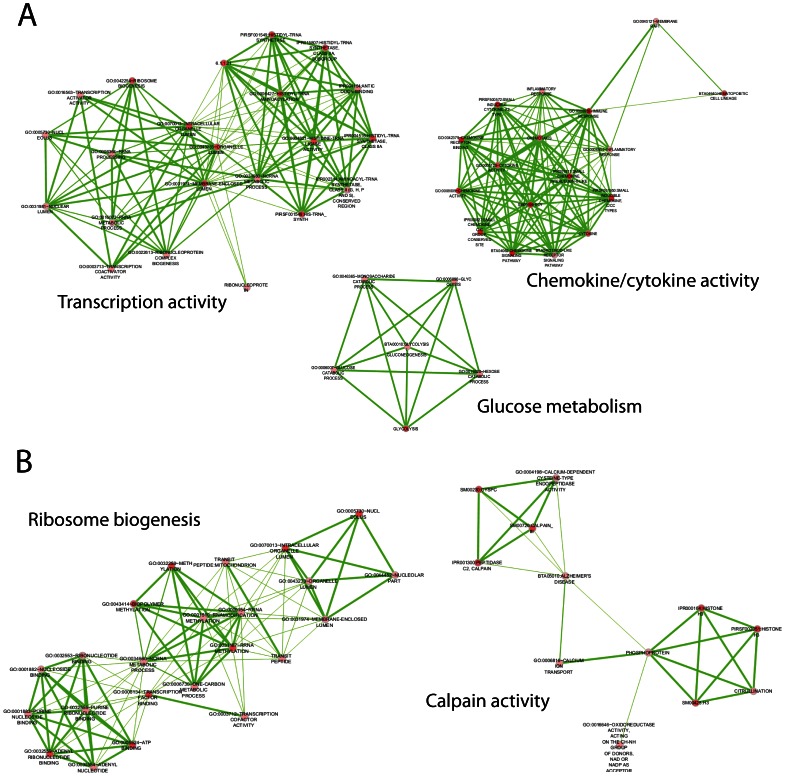
Descriptive Network of functional terms in Angus (A) and Brown Swiss (B). Nodes (red circles) are annotated functional terms. Edges connecting nodes represent gene share, being thickness proportional to the number of genes shared between terms (i.e., the degree of gene set overlap).

Networking of functional terms from ANG gene list (**Figure 3A**) revealed three groups: 1) immune response related genes, involved with chemokine and cytokine activity; 2) transcription activity, comprising the biosynthesis of ribonucleoproteins, transcription activation and aminoacylation of tRNA with L-histidine residuals; and 3) glucolysis and gluconeogenesis pathways. For BSW, a network related to post-transcriptional modifications of rRNAs (mostly methylation of adenosine residuals) and another involved with Calpain (**Figure 3B**) were observed. A significant intronic SNP (BTA16:27801014, *P* = 2.61×10^−7^) was detected in the Calpain 2 (*m-Calpain* - ENSBTAG00000012778) catalytic subunit, which may be capturing the signal of a causal untyped variant under selection. Due to a low number of genes mapped, it was not possible to build a network of functional terms for GYR and NEL. Across all lists, a total of 69 genes (13.69%) had no functional term associated to them, being either uncharacterized proteins or novel RNAs with no functional record available. All *DAVID* annotation chart reports are provided in **File S3**.

## Discussion

Concordances among *EHH* based tests seemed to have led the composite statistics in most cases, and disagreements between *Rsb* and *iHS* scores showed severe drop in significance support. It was noticed that *SHp* and Δ*DAF* did not contribute much towards spatial resolution individually, but they did help pinpointing SNP when blended with the other methods. This was in line with observations made by [6] when applying the Δ*DAF* method, which despite the little power to localize the sweep alone, showed to better distinguish selected from neutral variants in that study. All methods applied in this study were deemed capable of identifying recent sweeps, as well as signatures dating back up to a few thousand generations [2]. Considering that the significance of the combined test was mainly influenced by *EHH* based tests, and that cattle generation interval vary between 3 and 5 years, the methodology applied could have identified sweeps that happened as far as 6,000 years ago (1,200 cattle generations). Although this comprises most of cattle domestication history, the majority of the signals detected are more likely to have arisen during breed formation, which goes up to some hundreds of years ago [30]. This argument is based on two observations: 1) the meta-analysis method applied herein focused on breed-by-breed test integration, which may have favored the detection of breed-specific recent signatures; 2) for strong positive selective sweeps, which may have happened early in cattle domestication, the favored allele is expected to be nearly fixed across cattle breeds, and intrinsic factors of the present study contributed to the underrepresentation of fixed loci within the dataset used.

One factor that contributed to the underrepresentation of fixed loci in the dataset used is related to the SNP assay. As the SNP ascertainment strategies for the design of bovine arrays were focused on developing marker panels of common variations to support genome-wide association applications, and relied on sequence data of most major breeds for variation detection [28], the presence of SNP sites harboring rare variants (i.e. nearly fixed loci across cattle breeds) is scarce. Even if sequence data was used, the bovine reference genome available for detecting variants is the domesticated type [31], and sites of variation that underwent strong positive selection during domestication are probably difficult to be identified, as the unselected variant may be very rare. For instance, Rubin *et al*. (2010) [4] sought genome-wide heterozygosity depression in chicken using low coverage whole genome sequence data of DNA pools of domestic and wild lines, and the reference genome of what is considered to be the ancestral type (*Gallus gallus*). The strategy allowed for the detection of genome regions that were nearly fixed in the domesticated lines and exhibited low identity to the wild-ancestor haplotypes, suggesting selection sweeps during domestication.

Another important factor contributing to the low representation of rare variants in the present study is the filtering of SNP with moderate allele frequencies in all breeds (MAF ≥0.03), which may have made the detection of selection sweeps dating back to early cattle domestication unlikely. Nevertheless, the strategies adopted seemed to be capable of detecting footprints of recent positive selection, which may be anthropogenically ascertained by breeding and related to milk and meat production. The functional findings discussed later support this hypothesis.

**Figure 4:**
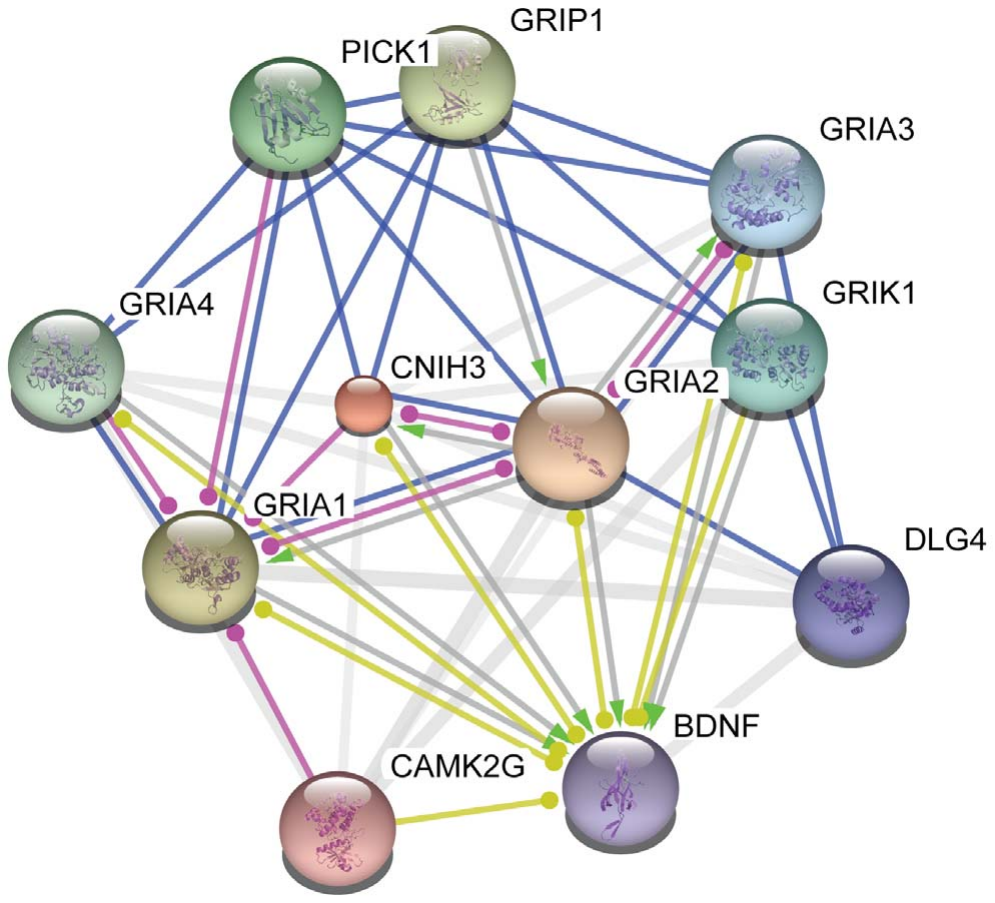
Protein network of human CNIH3, according to STRING 9.0 action view. Nodes are proteins; edges and arrows indicate interaction. Blue edges: binding; green arrows: activation; pink edges: post-translational modification; yellow edges: expression.

The most significant signal found comes from the *CNIH3* gene in BSW (*P* = 3.82×10^−12^). **Figure 4** shows all present date known and predicted relationships of human *CNIH3* with other proteins assessed by data integration in the *STRING 9.0* database [32], which indicates direct interaction of *CNIH3* with multiple α-amino-3-hydroxy-5-methyl-4-isoxazolepropionic acid (AMPA) selective glutamate receptors (*GRIA1*, *GRIA2*, *GRIA3*, *GRIA4* and *GRIK1*). *CNIH3* regulates the trafficking and gating properties of AMPA receptors in the central nervous system [33], which were previously shown to participate in luteinizing hormone (LH) secretion [34]. Sugimoto *et al*. (2010) [35] detected a single amino acid substitution (Ser -> Asn) in the bovine GRIA1 that leads to decreased release of gonadotropin-releasing hormone (*GnRH*) and slower pre-ovulatory *LH* surge, making carrier cows less responsive to superovulation hormone treatment. Sugimoto *et al*. (2010) [35] sequenced *GRIA1* in Japanese Black and Holstein commercial sires and found no departures from HWE in the locus, meaning that there is no evidence of selection pressure on the reported variants in either breeds. The signal on *CNIH3* found in the present study suggests that at least the underlying pathway has suffered recent selection pressure in BSW, although the selection force is unknown.

ANG exhibited three groups in the network map, one addressing chemokine/cytokine activity, a second with components of the transcription machinery and another related to glucolysis/gluconeogenesis. Both transcription activity and glucose metabolism are broad themes to be hypothesized, but it is possible that they have faced recent selection for high metabolic efficiency relative to increased meat yield and fat deposition. Regarding cytokines and chemokines, it has been found that they modulate different stages of muscle cell development [36], [37]. In a recent work, Zhao and collaborators (2012) [38] found evidence that RNA expression of genes involved with acute inflammatory response has high influence in meat tenderness in Angus cattle. They observed that chemokines and cytokines genes, including *chemokine C-C ligand 8* present in our gene list (ENSBTAG00000014113, BTA19), were deregulated in animals submitted to a surgical procedure, which in turn showed higher Warner-Bratzler shear force in beef samples after slaughter compared to the control group, suggesting that they play important role in muscle metabolism, either *in vivo* or in *postmortem* proteolysis regulation. These findings support that genes participating in chemokine/cytokine activity are under selection in ANG cattle.

The calpain-calpastatin system is a proteolytic complex that has also been largely incriminated in *postmortem* meat tenderization in beef cattle [39]. However, evidence of selection for components related to this system in the BSW data is somewhat surprising. Based on proteome analysis, Kuhla *et al*. (2011) [40] proposed a model in which the muscle breakdown provides substrates for milk production in early lactation, being a key mechanism in the nutritional imbalance of high-yielding dairy cows. Although [40] did not mention the calpain-calpastatin system, this may be one hypothesis for the overrepresentation of related terms found. Alternatively, Arnandis *et al*. (2012) [41] has shown that calpains are responsible for mitochondrial and lysosomal membrane permeabilization during lysosomal-mediated mammary epithelial cell death in mice. Also, milk yield is known to decline as function of many factors after peak lactation, including decrease in alveolar secretory epithelial cell number due to programmed cell death [42]. These evidences, together with the functional terms found, bring a second hypothesis that calpain-related genes are candidates under selection for lagged or mild post-peak lactation mammary gland involution in BSW. Both hypotheses point to the calpain-calpastatin system as a new target pathway involved in lactation dynamics in dairy cows.

Another intriguing candidate pathway pointed out by the present study is the ribosome biogenesis in BSW, more particularly the step involving methylation of rRNA. The addition of a methyl group to the 2′-hydroxyl group of the backbone ribose is a conserved type of post-transcriptional RNA modification [43], and is an essential step in ribosome assembly. Most 2′-O-methylated sites occur in functionally important regions of rRNAs and may influence ribosome structure and function [44]. It has been found that expression of ribosome components did not increase, and some of them had a slight decrease, during lactation in bovine mammary, which may be due to prioritization of synthesis of milk-specific mRNAs [45]. Thus, since the anabolic demand in lactation is not accompanied by increase in the expression of ribosome components, rRNA post-transcriptional modifications may play an important role in the translation efficiency of milk-specific proteins during lactation.

It was found an intronic signal for *ST6GALNAC5* (ENSBTAG00000007309) in GYR (*P* = 1.24×10^−9^). *ST6GALNAC5* is involved in the synthesis of gangliosides, more particularly the GD1α in the brain [46]. Gangliosides are glycosphingolipids containing one or more sialic acid residues in their structure, mainly n-acetylneuraminic acids. Some types of gangliosides can be found as components of the membrane fraction of the milk fat globule, which derives from the apical plasma membrane of secretory cells in the lactating mammary gland [47]. Prolactin-dependent deposition of GD1α gangliosides in the milk fat globules of mice (comprising up to 80.5% of the total milk lipid-bound sialic acid at the 3rd day of lactation) has been reported as a result of the expression of *ST6GALNAC5* during lactation, and may be an important source of GD1α for the developing neonate brain [46]. These evidences raise the hypothesis that *ST6GALNAC5* has been indirectly selected in GYR via percentage of fat in the milk.

The present study found substantially fewer evidences of recent selection in GYR and NEL, relative to BSW and ANG. When only within population tests were combined in the *meta-SS* statistics, GYR and NEL exhibited considerable numbers of selective sweeps, but still less than the taurine breeds analyzed (**Figure S3**). However, when only between populations tests were combined, the indicine breeds showed a severe drop in signals (**Figure S4**). As tests based on LD persistency and unusual long-range haplotypes were an important part of the composite statistics, the decreased number of sweeps found in the indicine breeds could be explained by differences in haplotype block structure and extent of LD across taurine and indicine breeds. In fact, ANG and BSW were shown to have greater mean haplotype block size and average LD than GYR and NEL [48]. Thus, the higher extended haplotype homozygosity of taurine breeds may have masked the detection of selective sweeps in the genomes of indicine breeds.

Many studies on signatures of selection in cattle have been published in recent years, and known genomic regions under selection are often used in literature as ‘confirmatory’ loci in order to validate new findings. Examples of such loci are the *melanocortin 1 receptor* gene (*MC1R* - ENSBTAG00000023731), responsible for the black/red coat color in ANG [28], [49], [50], and the *Mast/stem cell growth factor receptor* gene (*KIT* - ENSBTAG00000002699), incriminated in the ‘piebald’ spotted coat-color in Hereford and BSW [50], [51]. *MC1R* is located at BTA18: 14757332-14759082, and *KIT* is located at BTA6:71796318-71917431 in the UMD v3.1 assembly. Both *KIT* and *MC1R* regions were underrepresented in SNP coverage in the present study due to QC effects and ancestral allele information availability, and gaps spanning BTA6 71.7–72.4 Mb and BTA18 14.0–15.0 Mb were observed. These observations could justify the absence of significant signals for *KIT* or *MC1R*. However, other studies searching for selective sweeps in these breeds also did not report signals in *KIT* and *MC1R* regions in BSW and ANG [29], [52], respectively.

Some of the putative loci under selection detected herein were compared to previous studies, more particularly the signals found in BSW, since information on signatures of selection is more abundant in taurine dairy cattle. The topology of iHS -log10(*P*-values) across BTA 4, 5, 16 and 19 reported by [53] was noticeably similar to the *meta-SS* reported herein, and BTA 6 exhibited similarities with iHS reported by [52], [53] in Brown Swiss and by [54] in Norwegian Red. Hayes *et al*. (2008) [55] assessed evidence of divergent selection in Holstein and Angus using F_ST_ and iHS, and were able to detect signals in Holstein BTA 6 that resembled the *meta-SS* pattern found in the BSW dataset used in the present work. Moreover, Flori *et al*. (2009) [56] examined F_ST_ within and across three French dairy cattle breeds, finding putative regions under selection that overlap the findings on BSW chromosomes 5 and 6 in the present study.

Based on the findings presented, the combination of multiple methods and the functional annotation strategies adopted seemed to be highly informative. Notwithstanding, some challenges still need to be overcome when considering scanning genome-wide data for selection sweeps. First, as similar genomic patterns can be produced by other phenomena, such as genetic drift, separating false positives from real selection signals may not be trivial. Second, identified candidate regions often lacked spatial resolution, spanning from hundreds of kilobases to few megabases and comprising many genes. Third, distinguishing causal variants from nearby neutral loci may be the most difficult issue, as those variants were probably seldom typed in SNP arrays, and even with whole genome sequence data, variants in LD with the actual selected locus could have produced similar signals due to genetic hitch-hiking. Integrating different methodologies may help mitigating these problems, and should provide a valuable tool for seeking loci that are likely to have undergone recent artificial selection.

Finally, hypothesis making research implies proposing the function given the loci. In the present paper, this has meant inferring the source of selection for a given set of significant signals by extracting known gene functions and interaction information from available databases resources. Although the adopted functional annotation workflow using automated database mining and networking seemed to be a useful tool for providing insights on the driving forces behind the signals, the comprehensive nature of the annotation approach was expected to retrieve analysis artifacts due to systematic biases. Thus, hypothesis-driven investigations on the findings herein reported will contribute to elucidate which functions did undergo selection.

## Supporting Information

Figure S1 **Histogram for each individual standardized test score.**
(TIF)

Figure S2 **Pearson correlation between each individual test** ***Z*** **-transformed** ***P*** **-values.**
(TIF)

Figure S3 **Manhattan plots of genome-wide meta-SS –log10(** ***P*** **-values) combining within breeds tests only.**
(TIFF)

Figure S4 **Manhattan plots of genome-wide meta-SS –log10(** ***P*** **-values) combining between breeds tests only.**
(TIFF)

File S1 **Supporting methods: Cryptic relatedness control and functional annotation.**
(PDF)

File S2 **BovineHD ancestral allele information.**
(ZIP)

File S3 **List of significant intragenic SNP and annotation chart reports.**
(XLSX)

File S4 **Lists of genes potentially in linkage disequilibrium with putative SNP under selection in Angus, Brown Swiss, Gyr and Nellore.**
(ZIP)
