# Supplementary figures and images for: Detecting Loci under Recent Positive Selection in Dairy and Beef Cattle by Combining Different Genome-Wide Scan Methods
Source: PLoS One. 2013 May 16;8(5):e64280. doi: 10.1371/journal.pone.0064280 (PMC3655949; doi:10.1371/journal.pone.0064280)

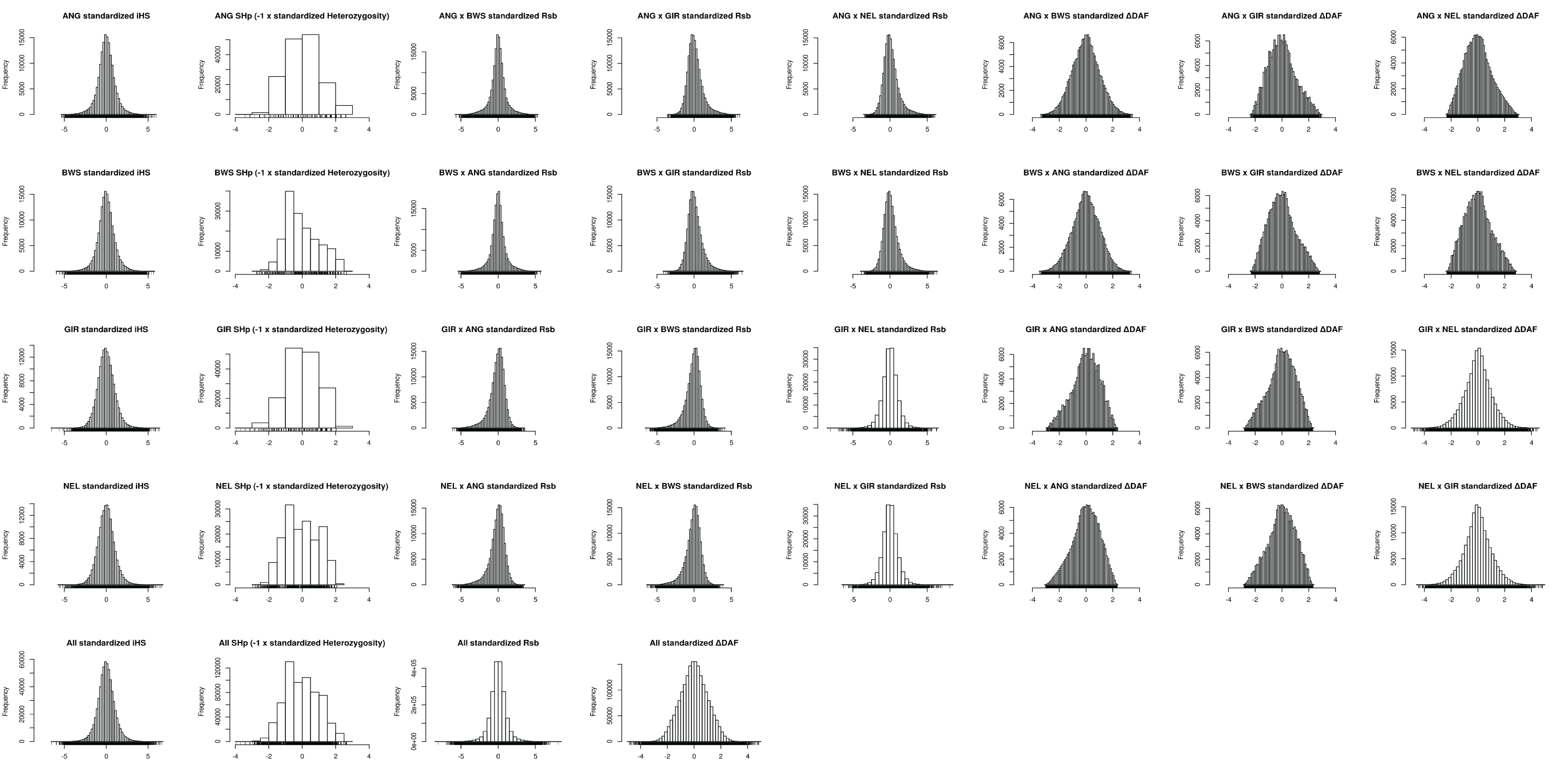

Supplement: Figure S1 — Histogram for each individual standardized test score. (TIF) [file pone.0064280.s001.tif]

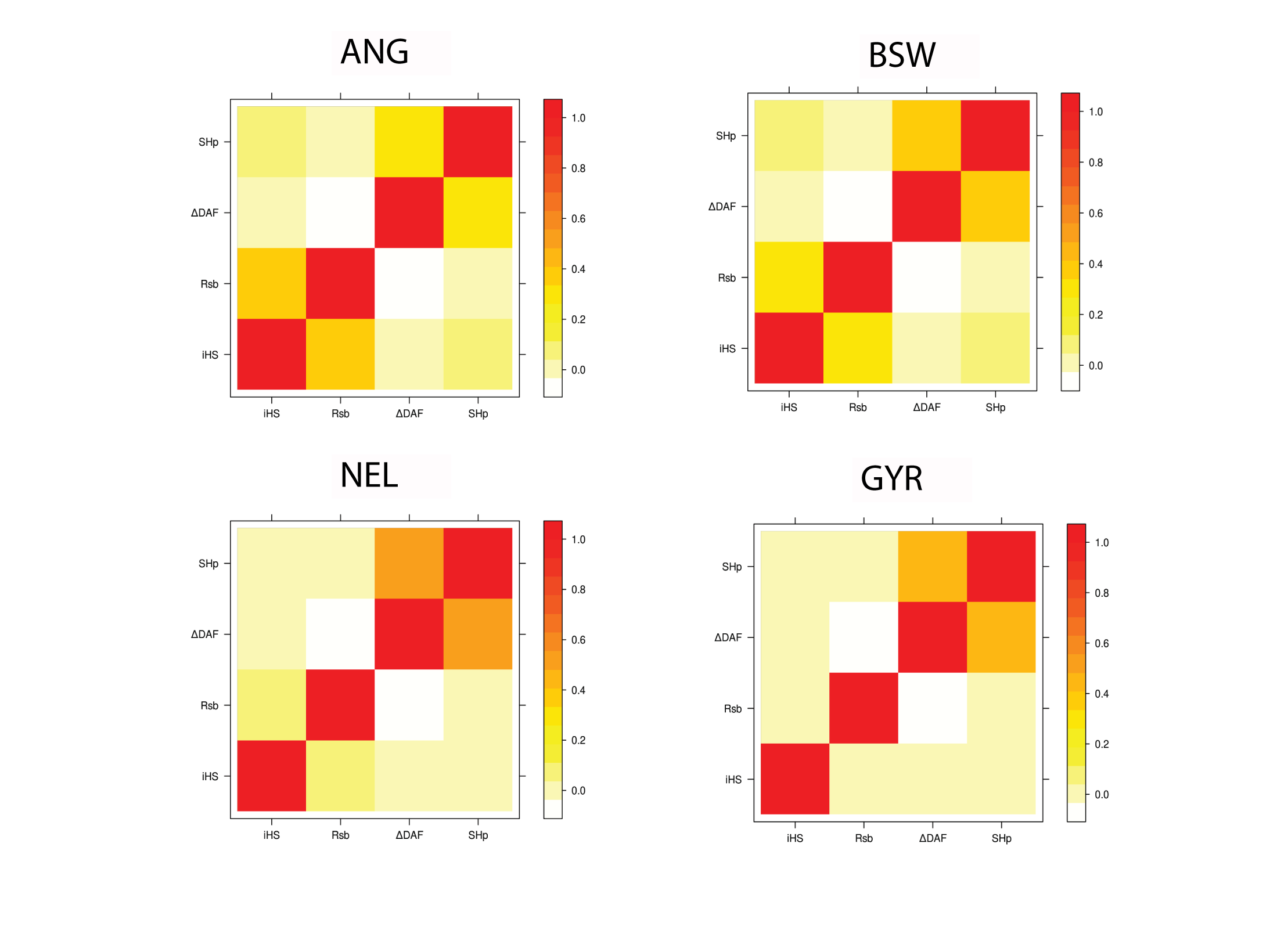

Supplement: Figure S2 — Pearson correlation between each individual test Z -transformed P -values. (TIF) [file pone.0064280.s002.tif]

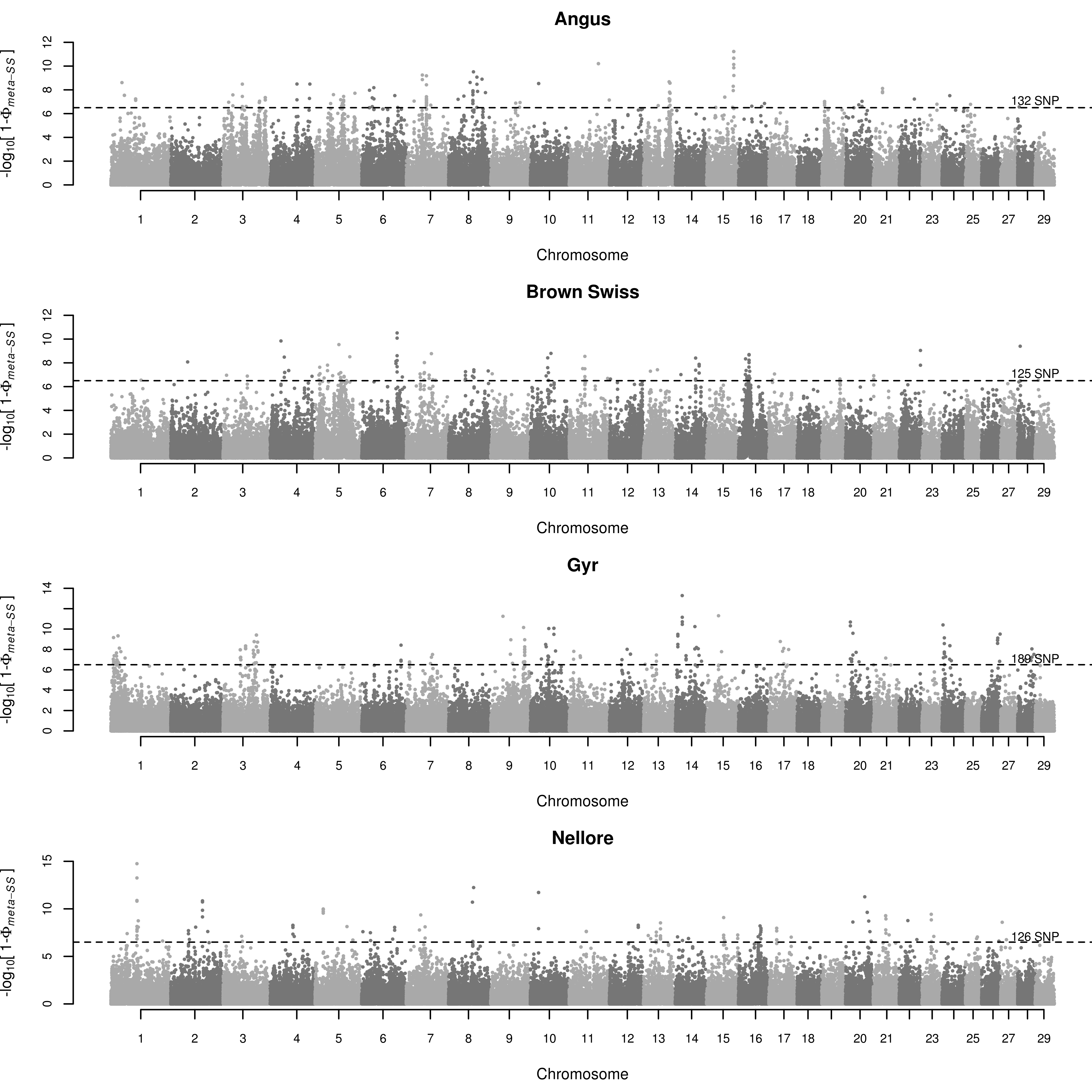

Supplement: Figure S3 — Manhattan plots of genome-wide meta-SS –log10( P -values) combining within breeds tests only. (TIFF) [file pone.0064280.s003.tiff]

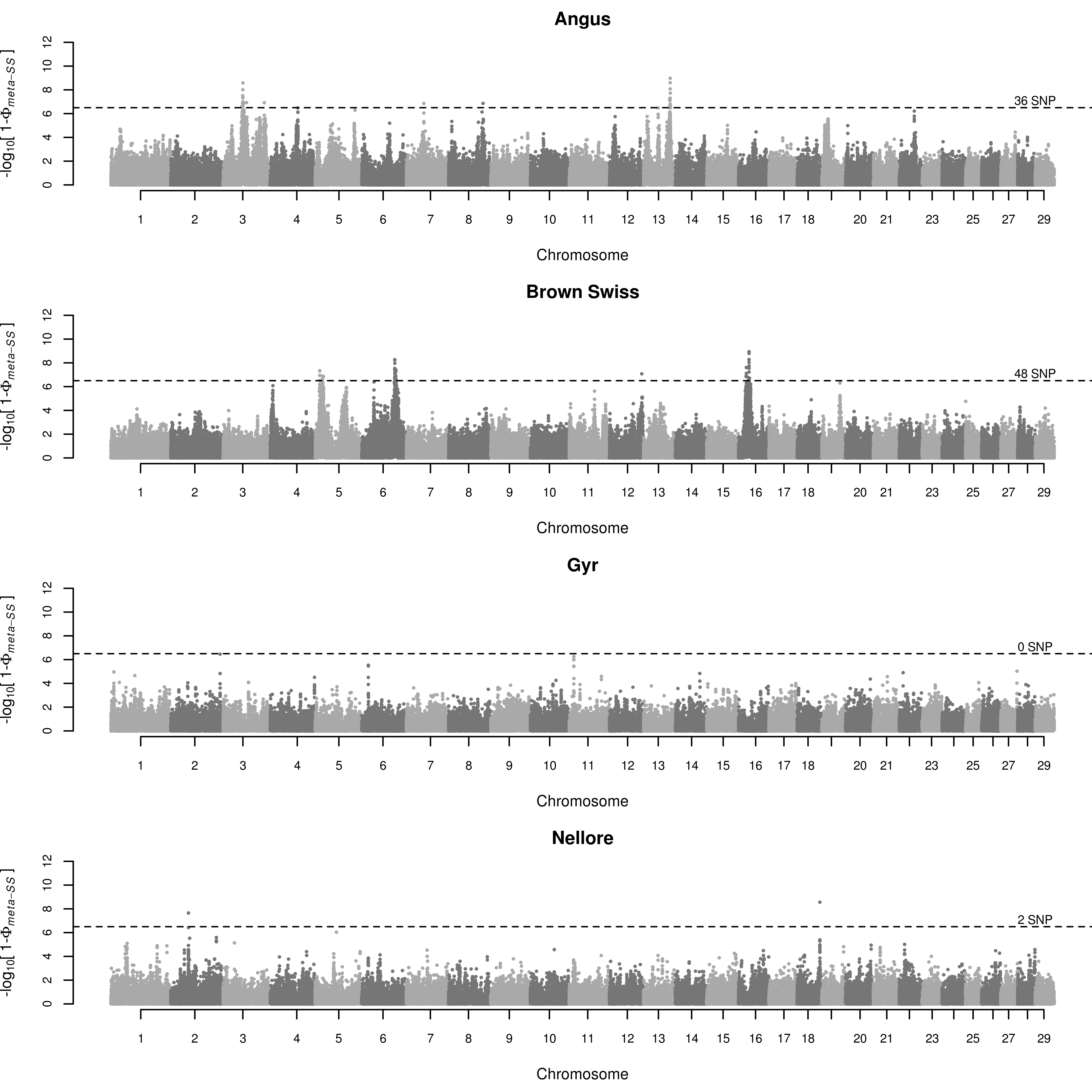

Supplement: Figure S4 — Manhattan plots of genome-wide meta-SS –log10( P -values) combining between breeds tests only. (TIFF) [file pone.0064280.s004.tiff]
