# Supplementary material for: Detecting Loci under Recent Positive Selection in Dairy and Beef Cattle by Combining Different Genome-Wide Scan Methods
Source: PLoS One. 2013 May 16;8(5):e64280. doi: 10.1371/journal.pone.0064280 (PMC3655949; doi:10.1371/journal.pone.0064280)
Supplement: File S1 — Supporting methods: Cryptic relatedness control and functional annotation. (PDF) [file pone.0064280.s005.pdf]

Supporting methods: Cryptic relatedness control and functional annotation

Cryptic relatedness control

We were interested in account for as much diversity as possible and keep only unrelated individuals within our dataset. It was known that BOKU and ZGC samples lodge different degrees of cryptic relatedness. According to The Bovine HapMap Consortium *et al*. (2009), their sampling strategy involved genotyping individuals that were unrelated for ≥ 4 generations, but each breed had at least one sire, dam and progeny trio. We expected to find duplicates within HapMap samples, because we knew the consortium had genotyped some animals twice for genotype quality assessment. It was also possible that there were duplicated samples between BOKU and HapMap or ZGC and HapMap, because high ranked sires may have been genotyped by the three initiatives. As our access to pedigree information was limited, we investigated our dataset for pairwise allele identity using *PLINK*. The method adopted is based on Identity by Descent (IBD) and was described by Purcell and collaborators (2007). Briefly, it uses a method-of-moments approach to estimate the probability of sharing 0, 1 or 2 alleles identical by descent for any two individuals, assuming they come from the same homogeneous, random-mating population. If we denote IBS states as *I* and IBD states as *Z* (in both cases, the possible states being 0, 1, and 2), then we can express the prior probability of IBS sharing as:

$$P(I = i) = \sum_{z=0}^{z=i} P(I = i \mid Z = z)P(Z = z)$$

As described in detail in Purcell et al (2007), for each SNP, the $P(I \mid Z)$ is specified in terms of the allele frequency; averaging over all SNPs, we obtain the expected global value for $P(I \mid Z)$. Then, rearranging the three equations implied by the equation above, we solve for $P(Z = 0)$, $P(Z = 1)$, and $P(Z = 2)$ and calculate:

$$\hat{\pi} = \frac{P(Z = 1)}{2} + P(Z = 2)$$

which is an estimation for the proportion of alleles shared identically by descent. Expected values of $\hat{\pi}$ for the different types of relationship can be found in **Table S1.1**. This estimate and its expected values were used for investigation of cryptic relatedness within breeds.

First, we looked for possible replicates. Duplicated (or even monozygote twins) samples usually present P(Z = 2) ~ 1 and $\hat{\pi}$ ~ 1 (i.e. all alleles are identical by descent). Thus, pairs of samples showing $\hat{\pi}$ ≥ 0.9 were considered duplicates, and one of the samples was randomly excluded. Second, heatmaps of $\hat{\pi}$ values were drawn for each breed, in order to obtain an overall view of the cryptic relatedness present within the datasets. To improve visualization even more, Euclidean distances were calculated from $\hat{\pi}$ dissimilarities and samples were clustered

according to the amount of alleles shared by descent. Finally, potential Parent-Offspring and Full-Siblings pairs were considered confounders for our analyses and at least one sample was excluded for each pair identified. As a single sample can hold first degree relationship with one or more samples at the same time, it is clear that its exclusion would solve the confounding effects and preserve more samples within the dataset than excluding random members of each pair. Thus, we developed an algorithm (written in R) that performs conservative exclusion of samples. For a given cryptic relatedness threshold (in this case, $\hat{\pi}$ > 0.4), the following procedure is executed:

a. For each sample, count the number of cryptic relationships it holds.

b. Sort samples by count score.

c. Exclude the sample with the highest score.

d. Repeat a, b and c until all scores are equal to 0.

Table S1.1. Different types of relatedness and their IBD values

Type	Degree	E($\hat{\pi}$)	$P(Z = 0)$	$P(Z = 1)$	$P(Z = 2)$
Full-Sibling	1-2	0.5000	0.2500	0.5000	0.2500
Half-Sibling	2	0.2500	0.5000	0.5000	0.0000
Grandparent-grandchild	2	0.2500	0.5000	0.5000	0.0000
Avuncular	2-3	0.2500	0.5000	0.5000	0.0000
First-Cousin	3	0.1250	0.7500	0.2500	0.0000
Unrelated	-	0.0000	1.0000	0.0000	0.0000
Half-Avuncular	3	0.1250	0.7500	0.2500	0.0000
Half-First-Cousin	4	0.0625	0.8750	0.1250	0.0000
Half-Sib+First-Cousin	2-3	0.3750	0.3750	0.5000	0.1250
Parent-Offspring	1	0.5000	0.0000	1.0000	0.0000
MZ-Twins	0	1.0000	0.0000	0.0000	1.0000

We found 7 BOKU-HapMap, 3 ZGC-HapMap and 3 HapMap-HapMap duplicates. After removal of replicated samples, we plotted the $\hat{\pi}$ heatmap (**Figure S1.1**) and carried out a principal component analysis (**Figure S1.2**) to check for the integrity of our genotype files and sample tracking. The number of remaining samples for each breed after duplicates and first degree relationship ($\hat{\pi}$ > 0.4) removal were: 24 ANG, 44 BSW (13 HapMap and 31 BOKU), 23 GYR and 581 NEL (24 HapMap and 557 ZGC). As NEL exhibited a sample size much larger than the other breeds, 45 individuals were sampled from the total 581 (all 24 remaining HapMap samples + 21 ramdom ZGC samples), in order to do fair comparisons. We decided to keep all possible HapMap genotypes because the sampling strategy adopted by the HapMap consortium attempted to account for as much within-breed diversity as possible.

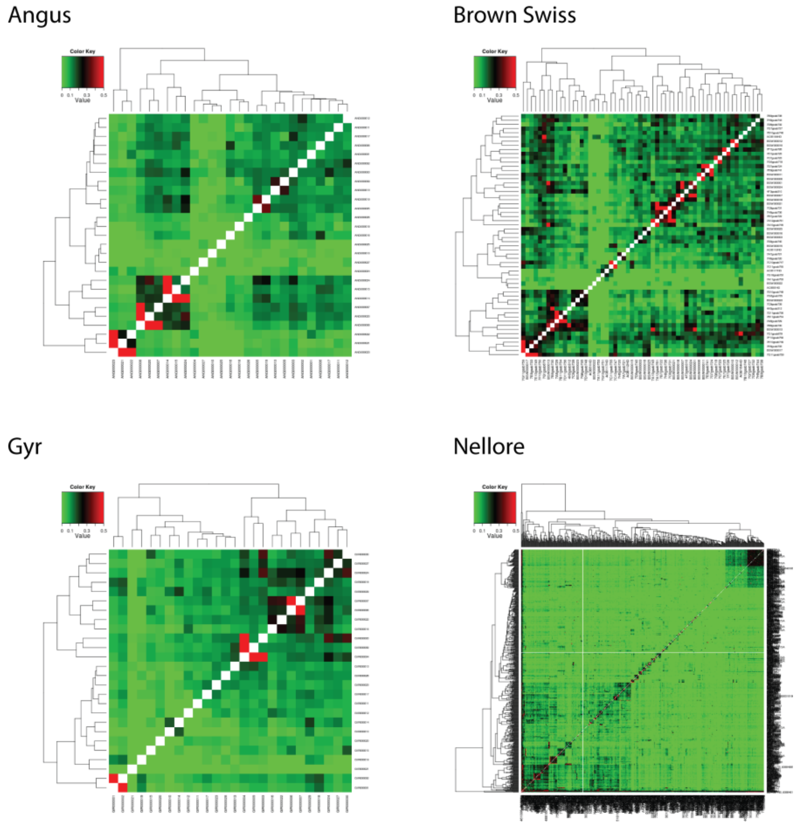

Figure S1.1 Heatmap and clustering of samples based on relatedness, as measured by $\hat{\pi}$. Values range from 0 (green - completely unrelated samples) to 0.5 (red - IBD sharing of half of the alleles, corresponding to Parent-Offspring or Full-Siblings pairs).

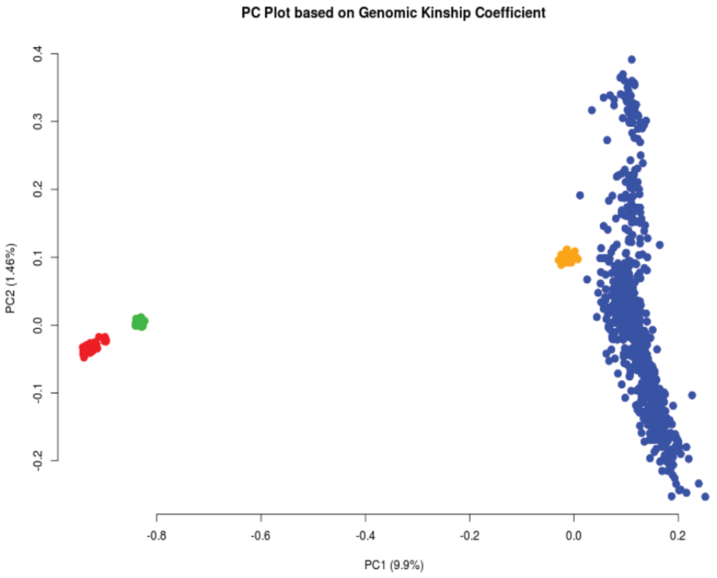

Figure S1.2 Principal Component Analysis. Red = BSW, Green = ANG, Orange = GYR and Blue = NEL. Percentages inside brackets correspond to proportion of variance explained by the respective eigenvectors.

Functional annotation

For any peak crossing the significance line, we applied three different strategies for the annotation of functional features.

Strategy 1: Since any given gene harboring signals is a direct candidate, the first approach consisted on checking if any significant SNP was intragenic via mining the *Ensembl Variation 67* database with the *Ensembl Biomart tool* (Kinsella *et al*., 2011).

Strategy 2: The closest gene in the vicinity of the most relevant SNP of a peak may be the responsible for the signal. Hence, the second strategy comprehended isolating the most significant SNP from each observed peak and mapping the closest gene to it. For that matter, we downloaded the Bovine UMD3.1 gene set from *Ensembl Genes 67* database via *Biomart tool* and used the *ClosestBed* algorithm from the *BedTools* software (Quinlan & Hall, 2010).

Strategy 3: There are cases where variants in multiple genes in linkage disequilibrium (LD) with the marker contribute to the signal together, because functionally related genes are often spatially close to each other. In fact, the usage of SNP chips is driven by the hypothesis that high marker density coverage of the genome is capable of capturing most of the genomic information by LD and haplotype structure. Thus, our third approach was a LD-based window scheme, divided into three steps.

Step 1: Every SNP crossing the significance line was defined as a 'core SNP'.

Step 2: We walked down to proximal and distal chromosome positions calculating correlations between the core SNP and the neighbor markers, checking if they tagged the core SNP or not based on r^2 values. The r^2 threshold adopted to declare that one marker tagged the core SNP was set to 0.7. The positions of the last tag markers on both sides of the core SNP, i.e., positions from where r^2 decayed below the defined threshold or the tag marker distance from the core position exceeded 1 Mb, were set as the boundaries of a window. This analysis was done in *PLINK*, using the options *--show-tags --list-all --tag-r2 0.7 --tag-kb 1000*.

Step 3: The retrieved window was interpreted as a single locus, and any gene overlapping it was considered to be in LD with the core SNP, thus a candidate for being involved with the selection signal. Such genes were therefore annotated. For the sake of marker density and region resolution, we used the lists of SNPs passing within breed QCs, regardless of ancestral allele information, instead of the unified list. For core SNPs where no window boundaries could be determined, we included the closest gene in the vicinity to the list. As some windows may also overlap, the derived gene list was then parsed to exclude repeated gene names and subsequently processed in

DAVID (Huang *et al*., 2009a; Huang *et al*., 2009b) for annotation of functional terms. We used the default parameters for each breed gene list, pooling together all genes annotated across the genome to reveal over-represented functional terms. Our hypothesis was not that all genome signals detected came from a single selection event, but that some sweeps may have shared the same functional background, i.e. the same selection force. Therefore, genes from different regions and chromosomes may cluster together or not based on their function, revealing biological processes, rather than single genes, that undergone selection. Finally, we used the *Enrichment Map Cytoscape plug-in* (Merico *et al*., 2010) to build networks of inter-related terms based on the number of overlapping genes. Terms were drawn as nodes (circles). Edges linking nodes represented gene sharing, and their thickness the degree of gene set overlap.

References

Huang, D.W., B.T. Sherman, R.A. Lempicki, 2009a Systematic and integrative analysis of large gene lists using DAVID Bioinformatics Resources. Nature Protoc 4(1):44-57.

Huang, D.W., B.T. Sherman, R.A. Lempicki, 2009b Bioinformatics enrichment tools: paths toward the comprehensive functional analysis of large gene lists. Nucleic Acids Res 37(1):1-13.

Kinsella, R.J., A. Kähäri, S. Haider, J. Zamora, G. Proctor *et al*, 2011 Ensembl BioMarts: a hub for data retrieval across taxonomic space. Database (Oxford). 2011 Published online Jul 23.

Merico, D., R. Isserlin, O. Stueker, A. Emili, G.D. Bader, 2010 Enrichment Map: A Network-Based Method for Gene-Set Enrichment Visualization and Interpretation. PLoS One 5(11):e13984.

Purcell, S., B. Neale, K. Todd-Brown, L. Thomas, M.A.R. Ferreira *et al*, 2007 PLINK: a toolset for whole-genome association and population-based linkage analysis. American Journal of Human Genetics 81(3):559-575.

Quinlan, A.R., I.M. Hall, 2010 BEDTools: a flexible suite of utilities for comparing genomic features. Bioinformatics 26(6):841-842.

The Bovine HapMap Consortium, R.A. Gibbs, J.F. Taylor, C.P. Van Tassell, W. Barendse *et al*, 2009. Genome-wide survey of SNP variation uncovers the genetic structure of cattle breeds. Science 324(5926):528-532.
